# Global trends in protected area connectivity from 2010 to 2018

**DOI:** 10.1016/j.biocon.2019.07.028

**Published:** 2019-10

**Authors:** Santiago Saura, Bastian Bertzky, Lucy Bastin, Luca Battistella, Andrea Mandrici, Grégoire Dubois

**Affiliations:** European Commission, Joint Research Centre (JRC), Ispra, Italy

**Keywords:** Aichi targets, Connectivity indicators, Protected areas, Protected area connectivity, Post-2020 biodiversity framework

## Abstract

Connectivity of protected areas (PAs) is needed to ensure the long-term persistence of biodiversity and ecosystem service delivery. The Convention on Biological Diversity agreed in 2010 to have 17% of land covered by well-connected PA systems by 2020 (Aichi Target 11). We here globally assess, for all countries, the trends in terrestrial PA connectivity every other year from 2010 to 2018 using the ProtConn indicator, which quantifies how well the PA systems are designed to support connectivity. The percentage of protected connected land (ProtConn) has increased globally from 6.5% in 2010 to 7.7% in 2018. Oceania experienced the largest recent increase in PA connectivity, whereas Asia is the only content with a lower ProtConn in 2018 than in 2010. Globally, the relative increase in the percentage of protected connected land (ProtConn) is nearly twice that of the percentage of land under protection (PA coverage), due to clear improvements in the design of PA systems for connectivity in many regions. The connectivity of the PA networks has become more dependent on the permeability of the unprotected landscape matrix in between PAs and on the coordinated management of adjacent PAs with different designations and of transboundary PA linkages. The relatively slow recent increase in PA connectivity globally (2016–2018) raises doubt as to whether connectivity targets will be met by 2020, and suggests that considerable further action is required to promote better-connected PA systems globally, including the expansion of the PA systems to cover key areas for connectivity in many countries and regions.

## Highlights

• We assess national and global trends (2010-2018) in the percentage of land that is protected and connected (ProtConn).
• ProtConn increased globally from 6.5% in 2010 to 7.7% in 2018, but it was higher in 2014.
• ProtConn increased much more than the percentage of land covered by protected areas (PAs).
• Recent trends suggest that global connectivity targets for PAs will not be met by 2020.
• Increased efforts are required on landscape permeability and on coordinated management of transboundary and adjacent PAs.

## 1 Introduction

Protected areas (PAs) need to be connected to meet their conservation goals (Ervin et al., 2010; Magris et al., 2018; UNEP-WCMC et al., 2018; UNEP, 2019). Poorly connected PA systems hamper species movements such as ranging, natal dispersal or seasonal migration, depending on species movement abilities and modalities. Species in isolated PAs suffer from the risks of inbreeding, extinction debt and reduced opportunities to adapt to climate change, all of which negatively impact species and genetic diversity, ecosystem health, and the long-term persistence of the biodiversity and ecosystem services provided by PAs (Newmark, 1996; DeFries et al., 2005; Heller and Zavaleta, 2009; Krosby et al., 2010; UNEP, 2019). This is well recognized in biodiversity and sustainability strategies and related targets at multiple levels (from local to global), such as the Aichi Targets of the Strategic Plan for Biodiversity 2011–2020 of the United Nations Convention on Biological Diversity (CBD, 2010). In particular, in Aichi Target 11 the international community agreed, in 2010, to increase the terrestrial area under protection to at least 17% in ‘effectively and equitably managed, ecologically representative and well-connected systems of protected areas’ by 2020 (CBD, 2010).

Despite the importance of PA connectivity for conservation goals, there have been very few quantitative assessments and indicators on how well connected PA systems are globally. Recently, Saura et al. (2018) provided the first assessment of global progress towards Aichi Target 11 element on well-connected terrestrial PA systems, using the Protected Connected (ProtConn) indicator. ProtConn is now one of the indicators included in the Biodiversity Indicator Partnership (BIP),^1^ which promotes and coordinates the development and delivery of biodiversity indicators for use by the CBD and other related conventions and frameworks. Saura et al. (2018) found that globally only 7.5% of the world's terrestrial surface was covered by protected and connected lands. However, Saura et al. (2018) provided only a single snapshot of PA connectivity, based on information on PAs as of 2016. To date, the temporal trends in PA connectivity since the Strategic Plan for Biodiversity was adopted in 2010 have not been assessed at global, regional and national levels, limiting the ability to measure the rate of progress towards Aichi Target 11.

Here we provide the first global assessment of trends in terrestrial PA connectivity from 2010 to 2018. We use the ProtConn indicator to assess, for each country, if PA systems are well designed to support connectivity. Specifically, we quantify the amount of protected land that is reachable in each country, focusing on the part of PA connectivity that is within the power of a country to influence, i.e. discounting the PA isolation due to the sea and to foreign lands. We use a reference median dispersal distance of 10 km, which is the central value of the log-transformed range of dispersal distances of most terrestrial vertebrates and captures well the global patterns of PA connectivity for other dispersal distances (Saura et al., 2017; Saura et al., 2018). Our assessment assumes that PAs are effectively managed and conserved to sustain connectivity, and that the landscape matrix outside PAs is unfavourable for movement, either because it may be currently degraded or because, given the lack of any protection, it may be converted to intensive land uses in the future, therefore not guaranteeing long-term support to a self-sufficient and well-connected system of PAs (Saura et al., 2018). We also identify the main strategic priorities for sustaining or improving PA connectivity in each country, which is relevant in guiding final efforts towards Aichi Target 11 as well as for other related post-2020 targets. We discuss how and why these priorities have progressively shifted since the Strategic Plan for Biodiversity was adopted in 2010.

## 2 Methods

### 2.1 Protected areas

We used the information on protected areas (PAs) provided by the World Database on Protected Areas (WDPA) for October 2010, June 2012, August 2014, June 2016 and June 2018 (IUCN and UNEP-WCMC, 2018). These dates were selected to cover the time from the adoption of the Strategic Plan for Biodiversity of the CBD in October 2010 to date, and to ensure consistency and comparability with previous global analyses on PAs that used the WDPA of June 2016 (Saura et al., 2018) or the WDPA of August 2014 (UNEP-WCMC and IUCN, 2014). The PA layer (WDPA) for each date was processed as in Saura et al. (2018) and further described in Appendix S1. In short, we removed all overlap between different PA designation types (topologically flat PA layer), and considered all PA polygons in the flat layer with a land area of at least 1 km^2^, which retained 99.8% of all land area under protection globally.

### 2.2 Countries

We assigned PAs to each country or territory based on the ISO3 country code reported in the WDPA. The ISO3 codes, to which we will here refer as ‘countries’ for brevity, correspond either to countries or to territories that are under the sovereignty of another country but are geographically distinct, such as Réunion Island, a French overseas territory located in the Indian Ocean. The use of ISO3 codes in the WDPA, and of the delineations of country boundaries and disputed territories in the GAUL layer for year 2015 developed by FAO^2^ (which we used in our maps below), does not imply any endorsement by the authors, nor any official position by the European Commission, on the sovereignty of these lands.

### 2.3 The protected connected indicator and its fractions

The Protected Connected (ProtConn) indicator (Saura et al., 2018) is defined as the percentage of a country that is covered by protected and connected lands. ProtConn can never be higher than PA coverage (percentage of the country covered by PAs, either connected or not), and will generally be lower because not all PAs are well connected to each other. We here use the indicator version named ProtConn_Bound_ in Saura et al. (2018). ProtConn_Bound_ focuses on the part of PA connectivity that is within the power of a country to influence, i.e. excluding PA isolation that is naturally imposed by the sea or that is due to foreign lands (out of the jurisdiction of the country). All analyses and results in this study use a reference median dispersal distance of 10 km (Saura et al., 2018). A negative exponential dispersal kernel is used, in which the probability of direct dispersal between PAs decreases in a continuous fashion as inter-PA distance increases (Saura et al., 2018). PAs separated by a distance equal to the median dispersal distance (10 km) have a probability of direct dispersal of 0.5.

In addition, we calculated the four fractions of ProtConn, expressed as a percentage of the total ProtConn value (Saura et al., 2017; Saura et al., 2018):

- ProtConn[Within]: percentage of the Protected Connected land that can be reached by moving only within the boundaries of individual PAs.
- ProtConn[Contig]: percentage of the Protected Connected land that can be reached by moving through sets of immediately adjacent (contiguous) PAs, without traversing any unprotected lands.
- ProtConn[Unprot]: percentage of the Protected Connected land that can be reached by moving through unprotected areas. It will be lower when PAs are separated by larger tracts of unprotected lands, making inter-PA movements less likely when compared to the median dispersal distance considered (10 km).
- ProtConn[Trans]: percentage of the Protected Connected land within the country that can be reached by moving through PAs located outside the country's boundaries. It does not measure the strength of the transboundary linkages but the degree to which the connectivity between PAs of the country depends on movement through PAs in other countries (the sources and destinations of the dispersal movement are PAs within the country).

ProtConn considers both intra-PA connectivity, and inter-PA connectivity, i.e. it accounts for both the amount of protected land that is available within individual PAs (as given by ProtConn[Within]) and that reachable by moving between different PAs (as captured by the other three ProtConn fractions). In this way, ProtConn acknowledges that the amount (or percent) of protected connected lands in a country may increase in two ways. First, through the designation of larger PAs, even if this results in a single PA that encompasses several previous smaller and well inter-connected PAs, as illustrated in Figs. A2 and A3 in Saura et al. (2018). Second, through more numerous or stronger connections between different PAs. Accounting for both intra-PA and inter-PA is necessary to provide a meaningful indicator of PA connectivity where increasing values result only from desirable conservation progress. In this way, for example, the indicator will decrease in response to the replacement of a PA by multiple smaller PAs covering a smaller proportion of the originally protected land (Saura et al., 2018).

We calculated ProtConn, its four fractions and the PA coverage for each of the considered years and for all countries, using the above-described PA layer. Based on these values, we determined the main strategic priorities for sustaining or improving PA connectivity in each country, as described in detail in Table 2 in Saura et al. (2018) and presented in the Results section below.

### 2.4 Indicator values at the regional, continental and global levels

We used the M49 standard of the United Nations Statistics Division^3^ to group countries into continents (called regions in M49) and regions (called sub-regions or intermediate regions in M49). We aggregated the indicator values for individual countries at the regional, continental and global level (excluding Antarctica) through area-weighted averages (Saura et al., 2018). The land area of the country was used as the weight for averaging the ProtConn values, which are expressed as a percentage of total land area of the country, while the product of the country land area and ProtConn was used as the weight for averaging the four ProtConn fractions, which are expressed as a percentage of the ProtConn value.

## 3 Results

**Fig. 1:**
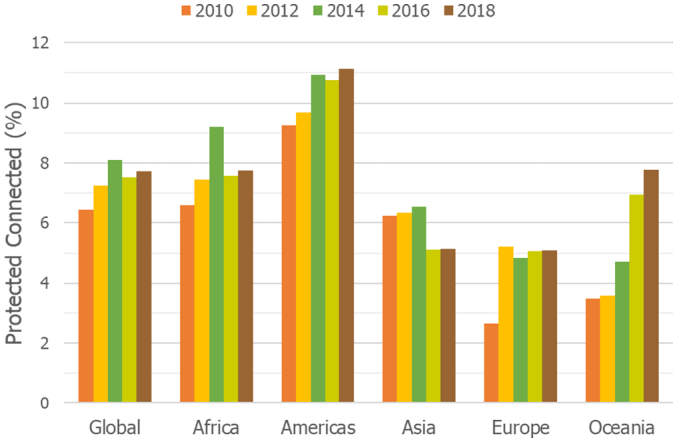
Trends in the percentage of protected and connected land (ProtConn) from 2010 to 2018 at the continental and global level. ProtConn values correspond to ProtConn_Bound_, which focuses on the part of PA connectivity that is within the power of a country to influence, i.e. excluding PA isolation that is naturally imposed by the sea or that is due to foreign lands (Saura et al., 2018). See Figs. B.1-B.5 in Appendix B for the ProtConn trends at the regional level.

**Fig. 2:**
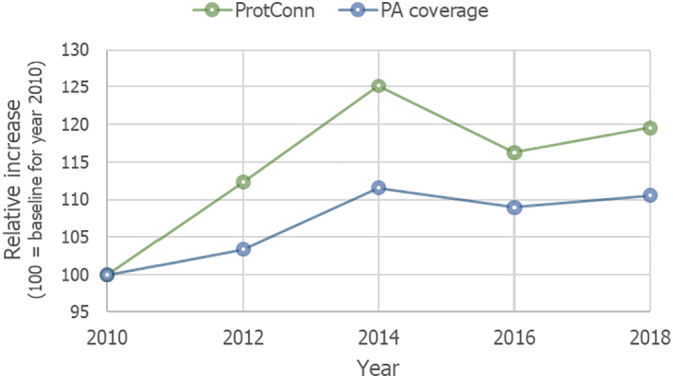
Relative variation in the global amount of protected land (PA coverage) and of protected and connected land (ProtConn) from 2010 to 2018. PA coverage and ProtConn are normalized so that 100 corresponds to their values in 2010. ProtConn values correspond to ProtConn_Bound_, which focuses on the part of PA connectivity that is within the power of a country to influence, i.e. excluding PA isolation that is naturally imposed by the sea or that is due to foreign lands (Saura et al., 2018).

The global percentage of protected and connected lands (ProtConn) increased from 6.47% in 2010 to 8.09% in 2014, then decreased to 7.52% in 2016 and again increased to 7.73% in 2018 (Fig. 1). As shown in Fig. 2, the relative increase in ProtConn from 2010 to 2018 was 19.5% globally (ProtConn increased from 6.47% to 7.73%), which is higher than the 10.5% of relative increase in PA coverage (percent of land covered by protected areas, whether connected or not), which increased from 13.50% in 2010 to 14.92% in 2018.

Oceania experienced the largest increase in PA connectivity from 2010 to 2018, which occurred mainly in recent years (after 2012), as shown in Fig. 1. ProtConn increases were also considerable in Europe (Fig. 1) and in the European Union (Fig. B.5), and particularly in Southern Europe (Fig. B.5). The European increase in ProtConn occurred almost entirely between 2010 and 2012 (Figs. 1 and B.5), and was strongly driven by the development of the Natura 2000 network in the European Union. The PA connectivity increase in the American continent as a whole (Fig. 1) was due entirely to South America and the Caribbean (Fig. B.2). All African regions except Northern Africa experienced an increase in ProtConn from 2010 to 2018 (Figs. 1 and B.1). Asia was the only continent with a lower ProtConn in 2018 than in 2010 (Fig. 1), which was largely due to PA degazettement in Western Asia (Fig. B.3).

**Fig. 3:**
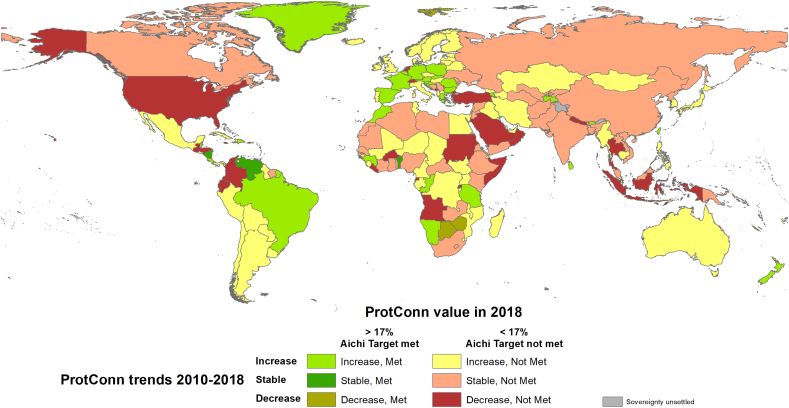
Trends in protected area connectivity (stable, increasing or decreasing) for all countries from 2010 to 2018 as quantified by the percentage of protected and connected land (ProtConn), and current status of the countries (as of 2018) regarding the connectivity element of Aichi Target 11, which is assumed to be met if ProtConn ≥ 17%. Countries here classified as with a stable trend have <0.2% of absolute difference in their ProtConn values in 2010 and 2018. India, for example, had a ProtConn of 1.15% in 2010 and a very similar ProtConn of 1.19% in 2018. ProtConn values correspond to ProtConn_Bound_, which focuses on the part of PA connectivity that is within the power of a country to influence, i.e. excluding PA isolation that is naturally imposed by the sea or that is due to foreign lands (Saura et al., 2018). See Figs. B.6 and B.7 in Appendix B for the same country classification using alternative thresholds different from 0.2% (0.1% and 0.5%) for the ‘stable’ classification.

**Fig. 4:**
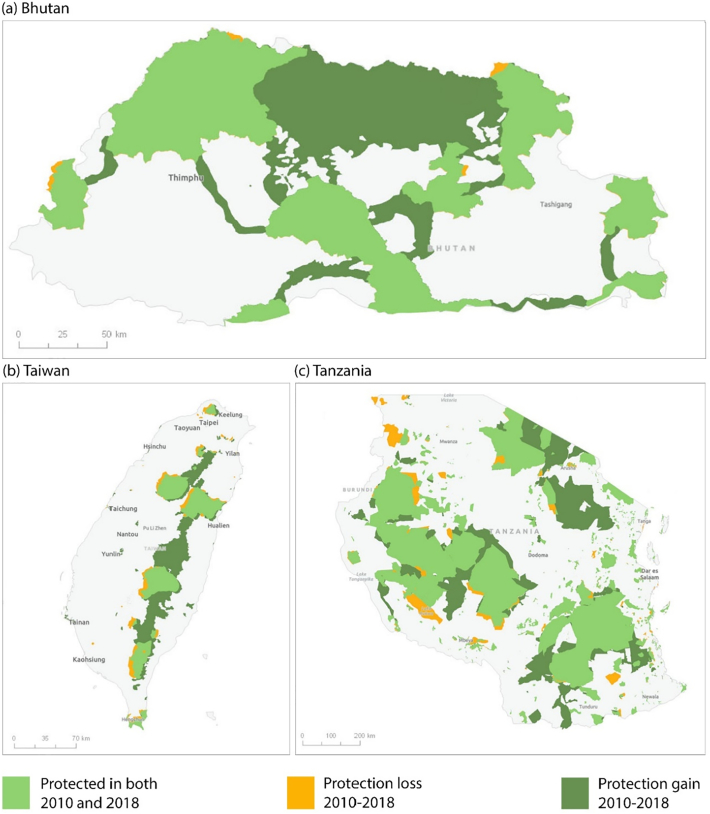
Three examples of countries or territories with notable increases in protected area connectivity from 2010 to 2018. In Bhutan (a), ProtConn increased from 16.9% in 2010 to 50.7% in 2018. In Taiwan (b), ProtConn increased from 6.2% in 2010 to 18.6% in 2018. In Tanzania (c), ProtConn increased from 16.4% in 2010 to 21.6% in 2018. The PA layers correspond to the dissolved PA layers used in this study, which do not show the boundaries between adjacent or overlapping PAs nor the PA polygons smaller than 1 km^2^. See Fig. B.9 in Appendix B for an additional example for Hungary.

About a quarter of the countries (28%), representing 43% of the global land area, had no notable change in PA connectivity during the last eight years (shown as ‘stable’ in Fig. 3), i.e. they had <0.2% of absolute difference in their ProtConn values in 2010 and 2018. This is the case of large countries like Canada, Russia or India, among others (Fig. 3). However, many countries experienced considerable ProtConn increases in the 2010–2018 period (Fig. B.8); Figs. 4 and B.9 provide some illustrative examples. Globally, the countries where ProtConn increased (56% of all countries) represented more than triple the number, and more than double the land area, of those where ProtConn declined (16% of all countries) (Fig. 3).

Countries such as Argentina, Australia and the Democratic Republic of Congo improved the connectivity of their PA systems from 2010 to 2018 but are still far from meeting the connectivity element of Aichi Target 11 (Figs. 3 and B.8), which is here assumed to be met when ProtConn≥17%. As of 2018, 70 countries, representing less than one third of all countries and only 14% of the global land area, meet the connectivity element of Aichi Target 11. This is almost double the 38 countries that met this target in 2010, which represented 5.7% of the global land area, but the same number of countries (70) as in 2016.

**Fig. 5:**
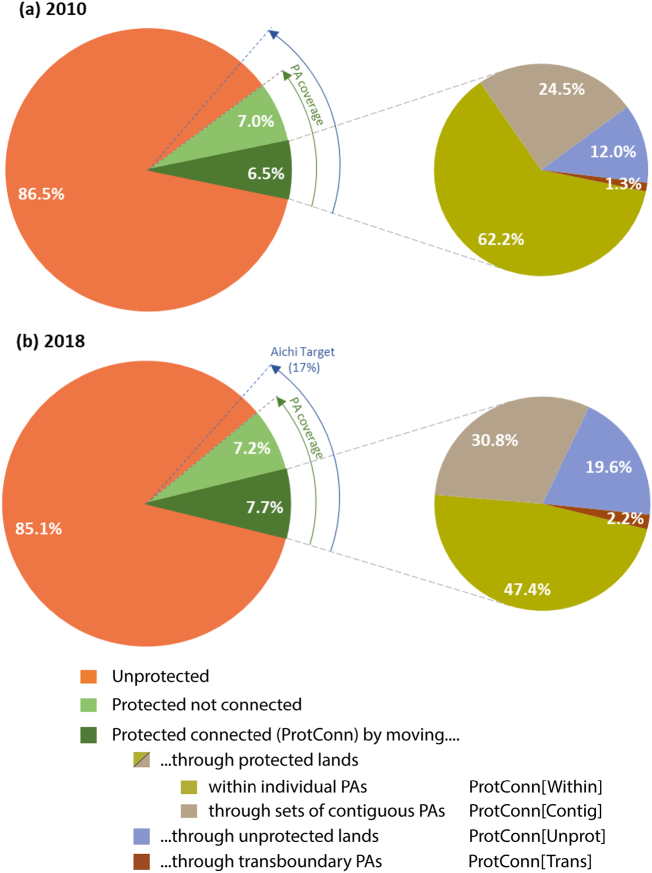
Global values of ProtConn, protected area (PA) coverage and the ProtConn fractions in 2010 (a) and 2018 (b). The global PA coverage in 2010 is 13.5% (6.5% + 7.0%), with the protected connected lands (ProtConn = 6.5%) representing a little less than half of the lands under protection. The global PA coverage in 2018 is 14.9% (7.7% + 7.2%), with protected connected lands (ProtConn = 7.7%) representing a little more than half of the lands under protection. ProtConn values correspond to ProtConn_Bound_, which focuses on the part of PA connectivity that is within the power of a country to influence, i.e. excluding PA isolation that is naturally imposed by the sea or that is due to foreign lands (Saura et al., 2018). The four ProtConn fractions assess the percentage of the protected connected land in a country that (i) can be reached within individual PAs: ProtConn[Within], (ii) can be reached by moving through adjacent PAs: ProtConn[Contig], (iii) depends on movement through unprotected lands: ProtConn[Unprot], (iv) depends on transnational linkages, i.e. on using PAs outside a country when moving between two PAs of the country: ProtConn[Trans].

From 2010 to 2018, globally, the amount of protected and connected land that could be reached by species became less dependent on individual PAs, as indicated by the lower values of ProtConn[Within] in Fig. 5. The connectivity of the PA networks became more dependent on the possibility of movement between adjacent PAs, on the permeability of the unprotected landscapes and on transboundary PA linkages, as indicated, respectively, by the higher values of ProtConn[Contig], ProtConn[Unprot] and ProtConn[Trans] in 2018 than in 2010 (Fig. 5).

**Fig. 6:**
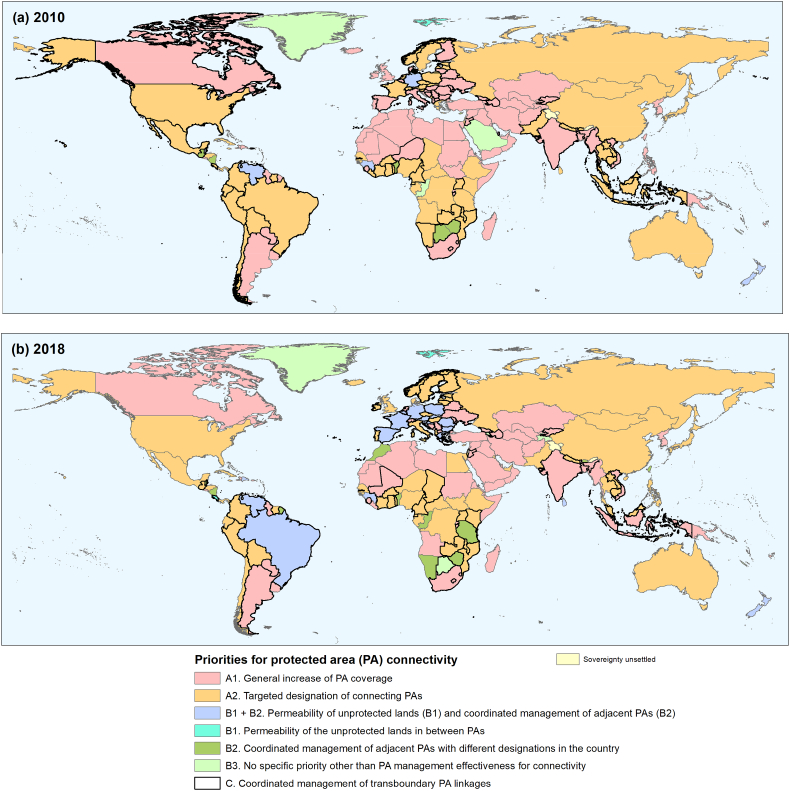
Main priorities for improving or sustaining protected area connectivity in each country considering the design of their protected area systems in 2010 (a) and 2018 (b), as given by the values of the ProtConn-related indicators. Effective protected area (PA) management for connectivity is an assumption of the ProtConn indicator (Saura et al., 2018), and is therefore a priority for all countries (and not just for those in B3).

The changes in the connectivity and design of PA systems, as indicated by ProtConn and its fractions, translated into shifts in the country-level priorities for sustaining or improving connectivity towards Aichi Target 11. In 2018 these priorities rely more frequently, compared to 2010, on efforts to ensure the permeability of the unprotected landscape matrix in between PAs and on the coordinated management for connectivity of adjacent PAs with different designations within the countries (Fig. 6). In 2018 these two priorities are the main ones for twice the number of countries, and for five times the total land area, than in 2010. The number of countries with a priority of new PA designation (priorities A1 and A2 in Fig. 6) has decreased slightly from 2010 to 2018, but this is still the main priority for about 72% of the countries in 2018, compared to 85% in 2010 (Fig. 6). Countries with this A1-A2 priority represent about 86% of the global land area, compared to 94% in 2010.

## 4 Discussion

The global protected area (PA) system is now significantly better designed for connectivity than it was in 2010. The higher gains in PA connectivity (ProtConn) than in the area under protection suggest that newly declared PAs have been able to play a significant role as corridors or stepping stones between existing PAs (see some remarkable examples in Figs. 4 and B.9). The notable PA connectivity gains from 2010 to 2014 were, however, followed by a decline from 2014 to 2016 and a mild increase from 2016 to 2018. The decline in ProtConn from 2014 to 2016 is due to a combination of two reasons. First, there have been some changes in the criteria used by some of the countries or territories to delineate and report PAs to the World Database on Protected Areas (WDPA). For example, a PA (‘Provinces du Sud’) of nearly 300,000 km^2^ covering the Western Sahara was reported by Morocco as a permanent hunting reserve in the WDPA of 2014 but was not present in the previous or later versions of the WDPA here considered. This led to a peak in the ProtConn values for Northern Africa in 2014, which more than doubled the values for the other dates in this region (Fig. B.1). Second, declines in ProtConn can result from actual changes in the capacity of national PA systems to support connectivity when governments renege or scale back on past commitments and designations. This includes cases of PA downsizing and degazettement (i.e. the elimination of protection for some previously protected areas), often associated with industrial-scale activities and local pressures largely decoupled from conservation objectives (Mascia and Pailler, 2011; Mascia et al., 2014; Dudley et al., 2018; Lewis et al., 2019), and often without any strategic compensatory designation of PAs elsewhere in the landscape. Even without an increase in PA degazettements, at the recent rates of PA connectivity increase here reported, it would take several decades to reach the level of 17% of protected and connected lands globally. This is because the absolute increase in ProtConn during the last 8-year period was 1.2% (from 6.5% in 2010 to 7.7% in 2018), while the shortfall to the 17% level is of 9.3% as of 2018. It is therefore unlikely that, given the current situation and trends of the global PA system, the connectivity element of Aichi Target 11 will be met by 2020.

There are however still opportunities to improve terrestrial PA connectivity levels up to 2020 and beyond. First, some PAs may exist for several years on the ground prior to being added to the WDPA,^4^ so that a given WDPA version does not contain all PAs gazetted to that date (Lewis et al., 2019). This lag effect may lead to some underestimate of PA connectivity in our assessment, which needs to be considered together with other gaps in the WDPA (Visconti et al., 2013). Second, many countries have committed to expand their PA systems to meet or approach Aichi Target 11 or regional and national targets; an additional 4.5 million km^2^ of land is expected to be designated as protected by 2020 (UNEP-WCMC et al., 2018). This is well in line with our indication that PA expansion efforts are the main priority to build well-connected PA systems in most countries (A1 and A2 in Fig. 6). The degree to which these committed or future efforts in PA expansion will contribute to reduce shortfalls in PA connectivity could be evaluated through the approach here presented, either at a scenario planning stage or after designation.

Aichi Target 11 (CBD, 2010) also foresees the recognition of and contributions from “other effective area based conservation measures” (OECMs) different from PAs, such as some territories and areas conserved by indigenous peoples and local communities (Gannon et al., 2017; Jonas et al., 2017; Dudley et al., 2018; Garnett et al., 2018; UNEP-WCMC et al., 2018), which have not been considered in this study. It is likely that OECMs will not only be helpful but essential in reaching connectivity targets and other conservation goals (Butchart et al., 2015; Gannon et al., 2017; Dudley et al., 2018). If well recognized, supported and managed, they could significantly enhance the connectivity of protected or conserved lands. Global and consistent data on OECMs is still lacking, but initiatives planned for the near future (UNEP-WCMC et al., 2018) will facilitate the assessment of their actual contribution to connectivity at a global scale. Conservation easements, conceived as a tool to protect private land from future development and intensive uses, may also provide an important contribution to PA connectivity and species migrations to be considered and quantified (Graves et al., 2019; Tack et al., 2019). The Connectivity Conservation Specialist Group of the World Commission on Protected Areas (International Union for Conservation of Nature)^5^ is working on guidelines for the recognition, establishment, spatial delineation and effective management of areas of connectivity conservation globally, which can contribute to significant progress in these directions.

The trends in PA connectivity and the changes in PA systems from 2010 to 2018 here reported highlight the increased importance of an international coordination of efforts towards well-connected PAs (Dudley et al., 2014). Globally, the connectivity of PAs in individual countries now depends almost twice as much on transnational connectivity compared to 2010 (Fig. 5). In the European Union (EU), the implementation of the EU-wide Natura 2000 network of PAs (Evans, 2012; EEA, 2015) has contributed to a remarkable improvement in the design of PA systems for connectivity from 2010 to 2012 (Figs. 1 and B.5), resulting in a broader transboundary PA system that is reflected in the priorities for most EU countries (see priority C in Fig. 6b). Similar cases can be found in other regions (Lysenko et al., 2007), such as the Kangchenjunga Landscape (shared by Bhutan, India and Nepal) and the Teraic Arc Landscape (shared by Nepal and India), which are considered some of the most important transboundary landscapes in the Himalayas (Chettri et al., 2007; Harihar and Pandav, 2012). Coordinated transnational management of PA connectivity is also important for many countries in Africa, in South and Southeastern Asia, and in South America (Fig. 6b), where eight countries are involved in the AAA (Andes-Amazon-Atlantic) Corridor project (e.g., Clerici et al., 2019). This transnational coordination can involve a range of initiatives and mechanisms: from government-led processes, such as signed memoranda of understanding or cross-border treaties, to informal collaboration agreements built around individual trust between managers or rangers across national boundaries (Dudley et al., 2014; Vasilijević et al., 2015).

Although, as noted above, PA expansion is a priority in most countries, some countries have significantly developed their PA systems since 2010, and now meet the 17% Aichi Target 11 target of protected and connected lands; for example, Brazil, New Zealand and several European countries (Figs. 3 and B.8). In these countries, improvements to PA connectivity now rely more on ensuring the permeability for species movements of the unprotected landscapes between PAs, and on the coordinated management of PAs with different designations in the country, rather than on the declaration of many new PAs (Fig. 6). This will require strategic action both at the PA planning level, going beyond management plans designed and implemented independently for individual PAs, and at the broader landscape planning level, involving mainstreamed actions across multiple sectors such as agriculture, transport infrastructure or urban planning (Donald and Evans, 2006; McDonald et al., 2009; Gurrutxaga et al., 2011; Güneralp and Seto, 2013; Zingg et al., 2019).

In conclusion, our study shows that despite the remarkable progress made by many countries since 2010 towards well-connected PA systems, considerable efforts remain necessary to meet global PA connectivity targets in the less than two years until the 2020 deadline of Aichi Target 11. Our assessment indicates where the shortfalls are concentrated globally and suggests the main priorities for further improvement, which may be of use for national, regional and global PA strategies. These insights, and our approach for monitoring PA connectivity are likely to remain relevant in the post-2020 biodiversity framework, where PA connectivity will play an important role and can be further improved with the support of regionally tailored strategies.

We emphasize, as noted in the Introduction, that ProtConn does not currently consider the heterogeneity of the unprotected landscape matrix between PAs. ProtConn only considers a PA to be well connected if it is well linked to other PAs. Functional connectivity that may be provided by an unprotected landscape with permeable land cover (e.g. primary vegetation) does not add to the ProtConn scores, because the lack of protection does not guarantee the maintenance of the connectivity role of this landscape in the long run. This means that, for example, if a connecting area that was formerly protected is degazzeted (unprotected), ProtConn will always decrease, no matter the land cover types that are present, in the short or long term, in this area. A different version of ProtConn may be however developed to account for the permeability of different land covers to species movements, so that the indicator is not only sensitive to changes in the design of the PA systems (as it currently is) but also to changes in the landscape matrix in between PAs. This would require from comparable time series of land cover, naturalness, landscape integrity or human pressures that can be used as proxies of the difficulty for species movements between PAs (Belote et al., 2016; Venter et al., 2016; Ayram et al., 2017; Kennedy et al., 2019). Extension of the ProtConn indicator to monitor connectivity trends in the marine realm would also be possible using the information on the WDPA for marine PAs; Engelhard et al. (2017) provide an interesting example of the application of similar underlying methods to evaluate seascape connectivity in a subtropical bay in Australia. Additional progress could be done by considering individual-based biophysical models that account for ocean circulation patterns not currently integrated in ProtConn or related indicators; Melià et al. (2016) provide an example of marine connectivity approaches that could inspire or enrich the development of ProtConn in this direction.

## Appendix A-B Supplementary data

Supplementary material Image 1

## Footnotes

1 https://www.bipindicators.net/indicators/protected-connected.

2 http://www.fao.org/geonetwork/srv/en/metadata.show?id=12691.

3 https://unstats.un.org/unsd/methodology/m49/.

4 https://www.protectedplanet.net/c/the-lag-effect-in-the-world-database-on-protected-areas.

5 https://www.iucn.org/commissions/world-commission-protected-areas/our-work/connectivity-conservation.

## References

[bb0005] Ayram C.A.C., Mendoza M.E., Etter A., Salicrup D.R.P. (2017). Anthropogenic impact on habitat connectivity: a multidimensional human footprint index evaluated in a highly biodiverse landscape of Mexico. Ecol. Indic..

[bb0010] Belote R.T., Dietz M.S., McRae B.H., Theobald D.M., McClure M.L., Irwin G.H., McKinley P.S., Gage J.A., Aplet G.H. (2016). Identifying corridors among large protected areas in the United States. PLoS One.

[bb0015] Butchart S.H., Clarke M., Smith R.J., Sykes R.E., Scharlemann J.P., Harfoot M. (2015). Shortfalls and solutions for meeting national and global conservation area targets. Conserv. Lett..

[bb0020] CBD (2010). Decision UNEP/CBD/COP/DEC/X/2 Adopted by the Conference of the Parties to the Convention on Biological Diversity at its Tenth Meeting. https://www.cbd.int/decision/cop/?id=12268.

[bb0025] Chettri N., Sharma E., Shakya B., Bajracharya B. (2007). Developing forested conservation corridors in the Kangchenjunga landscape, eastern Himalaya. Mt. Res. Dev..

[bb0030] Clerici N., Salazar C., Pardo-Díaz C., Jiggins C.D., Richardson J.E., Linares M. (2019). Peace in Colombia is a critical moment for Neotropical connectivity and conservation: save the northern Andes–Amazon biodiversity bridge. Conserv. Lett..

[bb0035] DeFries R., Hansen A., Newton A.C., Hansen M.C. (2005). Increasing isolation of protected areas in tropical forests over the past twenty years. Ecol. Appl..

[bb0040] Donald P.F., Evans A.D. (2006). Habitat connectivity and matrix restoration: the wider implications of agri-environment schemes. J. Appl. Ecol..

[bb0045] Dudley N., Groves C., Redford K.H., Stolton S. (2014). Where now for protected areas? Setting the stage for the 2014 World Parks Congress. Oryx.

[bb0050] Dudley N., Jonas H., Nelson F., Parrish J., Pyhälä A., Stolton S., Watson J.E. (2018). The essential role of other effective area-based conservation measures in achieving big bold conservation targets. Global Ecology and Conservation.

[bb0055] EEA (2015). State of Nature in the EU: Results from Reporting Under the Nature Directives 2007–2012. EEA (European Environment Agency) Technical Report No 2/2015. http://www.eea.europa.eu/publications/state-of-nature-in-the-eu.

[bb0060] Engelhard S.L., Huijbers C.M., Stewart-Koster B., Olds A.D., Schlacher T.A., Connolly R.M. (2017). Prioritising seascape connectivity in conservation using network analysis. J. Appl. Ecol..

[bb0065] Ervin J., Sekhran N., Dinu A., Gidda S., Vergeichik M., Mee J. (2010). Protected Areas for the 21st Century: Lessons from UNDP/GEF’s Portfolio.

[bb0070] Evans D. (2012). Building the European Union’s Natura 2000 network. Nature Conservation.

[bb0075] Gannon P., Seyoum-Edjigu E., Cooper D., Sandwith T., Ferreira de Souza Dias B., Pașca Palmer C., Lang B., Ervin J., Gidda S. (2017). Status and prospects for achieving Aichi Biodiversity Target 11: implications of national commitments and priority actions. Parks.

[bb0080] Garnett S.T., Burgess N.D., Fa J.E., Fernández-Llamazares Á., Molnár Z., Robinson C.J. (2018). A spatial overview of the global importance of indigenous lands for conservation. Nature Sustainability.

[bb0085] Graves R.A., Williamson M.A., Belote R.T., Brandt J.S. (2019). Quantifying the contribution of conservation easements to large-landscape conservation. Biol. Conserv..

[bb0090] Güneralp B., Seto K.C. (2013). Futures of global urban expansion: uncertainties and implications for biodiversity conservation. Environ. Res. Lett..

[bb0095] Gurrutxaga M., Rubio L., Saura S. (2011). Key connectors in protected forest area networks and the impact of highways: a transnational case study from the Cantabrian Range to the Western Alps. Landsc. Urban Plan..

[bb0100] Harihar A., Pandav B. (2012). Influence of connectivity, wild prey and disturbance on occupancy of tigers in the human-dominated Western Terai Arc Landscape. PLoS One.

[bb0105] Heller N.E., Zavaleta E.S. (2009). Biodiversity management in the face of climate change: a review of 22 years of recommendations. Biol. Conserv..

[bb0110] IUCN, UNEP-WCMC (2018). The World Database on Protected Areas (WDPA). http://www.protectedplanet.net.

[bb0115] Jonas H.D., Lee E., Jonas H.C., Matallana-Tobon C., Sander Wright K., Nelson F., Enns E. (2017). Will “other effective area-based conservation measures” increase recognition and support for ICCAs?. Parks.

[bb0120] Kennedy C.M., Oakleaf J.R., Theobald D.M., Baruch-Mordo S., Kiesecker J. (2019). Managing the middle: a shift in conservation priorities based on the global human modification gradient. Glob. Chang. Biol..

[bb0125] Krosby M., Tewksbury J., Haddad N.M., Hoekstra J. (2010). Ecological connectivity for a changing climate. Conserv. Biol..

[bb0130] Lewis E., MacSharry B., Juffe-Bignoli D., Harris N., Burrows G., Kingston N., Burgess N.D. (2019). Dynamics in the global protected-area estate since 2004. Conserv. Biol..

[bb0135] Lysenko I., Besançon C., Savy C. (2007). 2007 UNEP–WCMC Global List of Transboundary Protected Areas.

[bb0140] Magris R.A., Andrello M., Pressey R.L., Mouillot D., Dalongeville A., Jacobi M.N., Manel S. (2018). Biologically representative and well-connected marine reserves enhance biodiversity persistence in conservation planning. Conserv. Lett..

[bb0145] Mascia M.B., Pailler S. (2011). Protected area downgrading, downsizing, and degazettement (PADDD) and its conservation implications. Conserv. Lett..

[bb0150] Mascia M.B., Pailler S., Krithivasan R., Roshchanka V., Burns D., Mlotha M.J., Murray D.R., Peng N. (2014). Protected area downgrading, downsizing, and degazettement (PADDD) in Africa, Asia, and Latin America and the Caribbean, 1900–2010. Biol. Conserv..

[bb0155] McDonald R.I., Forman R.T., Kareiva P., Neugarten R., Salzer D., Fisher J. (2009). Urban effects, distance, and protected areas in an urbanizing world. Landsc. Urban Plan..

[bb0160] Melià P., Schiavina M., Rossetto M., Gatto M., Fraschetti S., Casagrandi R. (2016). Looking for hotspots of marine metacommunity connectivity: a methodological framework. Sci. Rep..

[bb0165] Newmark W.D. (1996). Insularization of Tanzanian parks and the local extinction of large mammals. Conserv. Biol..

[bb0170] Saura S., Bastin L., Battistella L., Mandrici A., Dubois G. (2017). Protected areas in the world’s ecoregions: how well connected are they?. Ecol. Indic..

[bb0175] Saura S., Bertzky B., Bastin L., Battistella L., Mandrici A., Dubois G. (2018). Protected area connectivity: shortfalls in global targets and country-level priorities. Biol. Conserv..

[bb0180] Tack J.D., Jakes A.F., Jones P.F., Smith J.T., Newton R.E., Martin B.H., Hebblewhite M., Naugle D.E. (2019). Beyond protected areas: private lands and public policy anchor intact pathways for multi-species wildlife migration. Biol. Conserv..

[bb0185] UNEP (2019). Frontiers 2018/19 Emerging Issues of Environmental Concern. https://www.unenvironment.org/resources/frontiers-201819-emerging-issues-environmental-concern.

[bb0190] UNEP-WCMC, IUCN (2014). Protected Planet Report 2014.

[bb0195] UNEP-WCMC, IUCN, NGS (2018). Protected Planet Report 2018.

[bb0200] Vasilijević M., Zunckel K., McKinney M., Erg B., Schoon M., Rosen Michel T. (2015). Transboundary Conservation: A Systematic and Integrated Approach.

[bb0205] Venter O., Sanderson E.W., Magrach A., Allan J.R., Beher J., Jones K.R., Possingham H.P., Laurance W.F., Wood P., Fekete B.M., Levy M.A. (2016). Sixteen years of change in the global terrestrial human footprint and implications for biodiversity conservation. Nat. Commun..

[bb0210] Visconti P., Di Marco M., Alvarez-Romero J.G., Januchowski-Hartley S.R., Pressey R.L., Weeks R., Rondinini C. (2013). Effects of errors and gaps in spatial data sets on assessment of conservation progress. Conserv. Biol..

[bb0215] Zingg S., Ritschard E., Arlettaz R., Humbert J. (2019). Increasing the proportion and quality of land under agri-environment schemes promotes birds and butterflies at the landscape scale. Biol. Conserv..

