# Supplementary material for: Global trends in protected area connectivity from 2010 to 2018
Source: Biol Conserv. 2019 Oct;238:108183. doi: 10.1016/j.biocon.2019.07.028 (PMC6919936; doi:10.1016/j.biocon.2019.07.028)
Supplement: Supplementary file 1 — Supplementary material [file mmc1.docx]

**Appendix A. Details on the processing of the protected area layers**

We processed the PA layer (WDPA) for each of the five dates as follows. First, we excluded PAs with a “proposed” or “not reported” status, PAs reported as points without an associated reported area, and UNESCO Man and the Biosphere Reserves. Second, we included, in addition to the PAs reported as polygons in the WDPA, those PAs provided in the WDPA as points (with unknown boundaries) but with a reported area, using a geodesic circular buffer with an area equal to the reported value. Third, we dissolved the PA layer by the ISO3 code of PAs to remove all overlaps between different designation types and avoid double counting (e.g. where the same area is designated both as a National Park and as a World Heritage Site) within each country or territory. Fourth, we excluded marine PAs and the marine portion of coastal PAs by using a land mask obtained from the Global Administrative Unit Layers (GAUL) for year 2015, developed by the Food and Agricultural Organization (FAO) of the United Nations^[[1]](#footnote-1)^. Fifth, we calculated the area of each resulting PA polygon and, for computational feasibility of the connectivity calculations, we removed those PA polygons with an area smaller than 1 km^2^, which retained 99.8% of the total land area covered by PAs globally. Sixth, in order to facilitate calculation of the inter-PA distance calculations, we reduced the number of vertices in the polygons using the Simplify Polygons tool in ArcGIS Pro with a tolerance of 0.001 decimal degrees (about 100 m at the equator). Seventh, we calculated the distances between all PAs as geodesic distances between the edges of the PAs, using the Generate Near Table tool in ArcGIS Pro. Inter-PA distances were calculated up to a maximum distance of 300 km, which included all PAs up to that distance from any PA of a given country, whether located in the same country or in some other neighboring nation. This allowed quantification of the transnational component of the ProtConn indicator (ProtConn[Trans]). The 300 km maximum distance was used because it is much larger than the median dispersal distance considered (10 km), and therefore includes all pairs of PAs between which dispersal movements may be likely. For the median dispersal distance of 10 km considered in this study, the probability of a dispersal movement between PAs separated by > 300 km is negligible (about 10^-8^) according to the negative exponential dispersal kernel used in the ProtConn calculations (Saura et al., 2018). Subsequent connectivity and data analysis were performed using the command line version of Conefor (Saura & Torné, 2009), available at [www.conefor.org](http://www.conefor.org), and R (R Core Team, 2019), respectively.

The versions of the World Database on Protected Areas (WDPA) used to produce the results in this study were, for all countries and territories, those mentioned in the main text, i.e. October 2010, June 2012, August 2014, June 2016 and June 2018. The only exception was made for China for year 2018. Since May 2018, China removed most of the Chinese PAs from the public version of the WDPA, although this does not mean that the PAs no longer exist; they have in general not been degazetted^[[2]](#footnote-2)^. The full set of Chinese PAs has only been available, since May 2018, in a restricted version of the WDPA that is used by UNEP-WCMC for monthly calculations of national PA coverage^[[3]](#footnote-3)^. For 2018, we therefore used the Chinese PA data from the public WDPA version of April 2018, to obtain all the results at the national (China), regional, continental and global level in this study. This avoided an unrealistic underestimation of the Chinese PA system (and the likely impact, given the large size of this country, on results at regional, continental and global scales) because of data restrictions which are not related to the actual status and design of the Chinese PA system. In this way, we obtained results for PA coverage in China and globally that are also consistent with those reported in a recent analysis using the restricted version of the WPDA as of July 2018 for China (UNEP-WCMC et al., 2018).

References cited in this appendix

R Core Team (2019). R: A language and environment for statistical computing. R Foundation for Statistical Computing, Vienna, Austria. Available at <http://www.R-project.org/>

Saura, S., & Torné, J. (2009). Conefor Sensinode 2.2: a software package for quantifying the importance of habitat patches for landscape connectivity. *Environmental Modelling and Software, 24*, 135–139.

Saura, S., Bertzky, B., Bastin, L., Battistella, L, Mandrici, A., & Dubois, G. (2018). Protected area connectivity: shortfalls in global targets and country-level priorities. *Biological Conservation, 219*, 53–67 https://doi.org/10.1016/j.biocon.2017.12.020

UNEP-WCMC, IUCN, & NGS. (2018). Protected Planet Report 2018. UNEP-WCMC, IUCN and NGS: Cambridge UK; Gland, Switzerland; and Washington, D.C., USA.

**Appendix B. Supplementary figures**

**
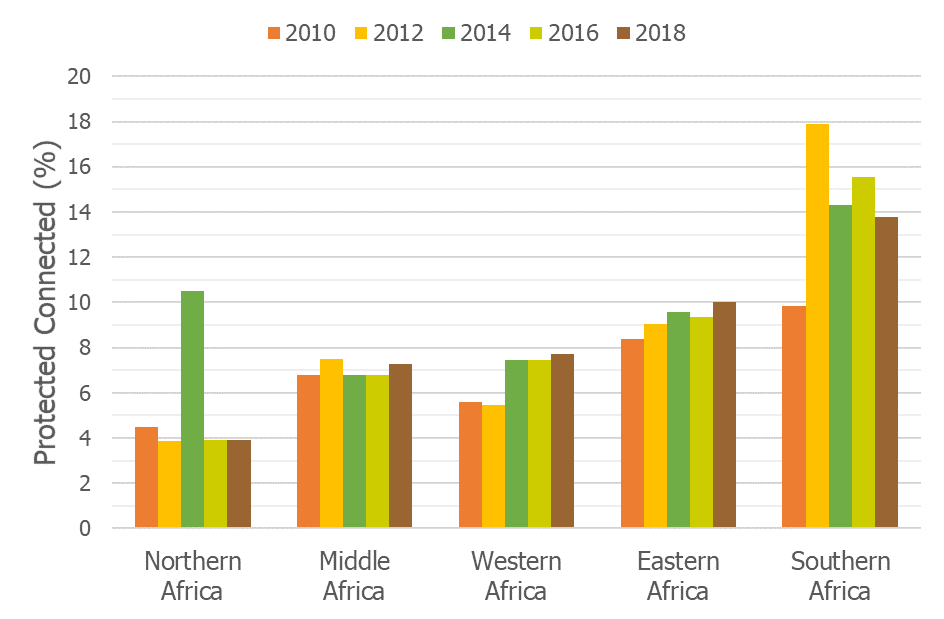
**

**Fig. B.1.** Trends in the percentage of protected and connected land (ProtConn) from 2010 to 2018 in the regions of Africa. ProtConn values correspond to ProtConn_Bound_, which focuses on the part of PA connectivity that is within the power of a country to influence, i.e. excluding PA isolation that is naturally imposed by the sea or that is due to foreign lands (Saura et al., 2018). Regions are defined according to the M49 standard of the United Nations Statistics Division (called sub-regions or intermediate regions in M49). The much higher ProtConn value for Northern Africa in 2014 than in any other year is largely due to the ‘Provinces du Sud’ PA reported by Morocco in the WDPA of 2014, which was not present in the previous or later versions of the WDPA here considered.

**
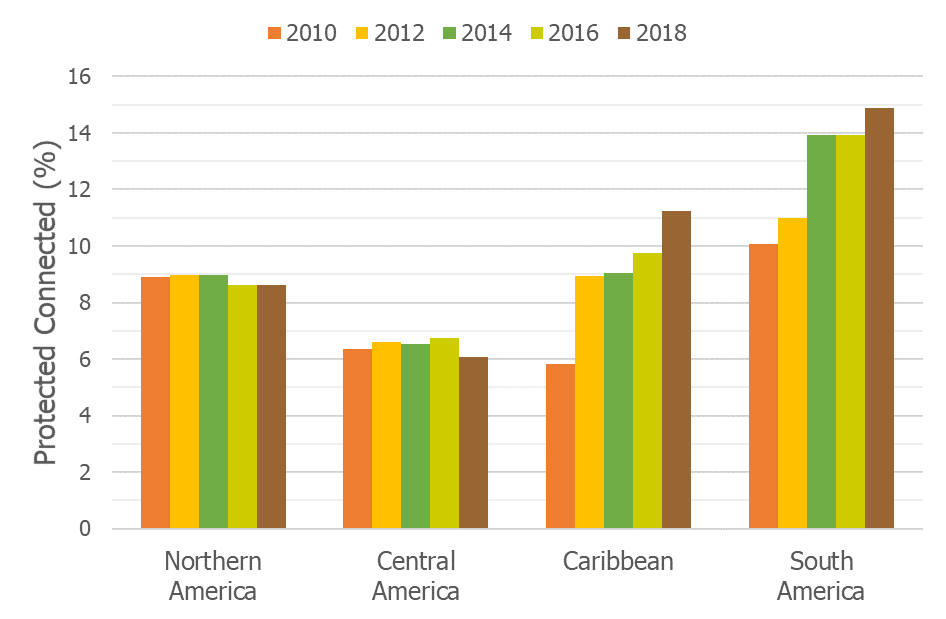
**

**Fig. B.2.** Trends in the percentage of protected and connected land (ProtConn) from 2010 to 2018 in the regions of America. ProtConn values correspond to ProtConn_Bound_, which focuses on the part of PA connectivity that is within the power of a country to influence, i.e. excluding PA isolation that is naturally imposed by the sea or that is due to foreign lands (Saura et al., 2018). Regions are defined according to the M49 standard of the United Nations Statistics Division (called sub-regions or intermediate regions in M49).

**
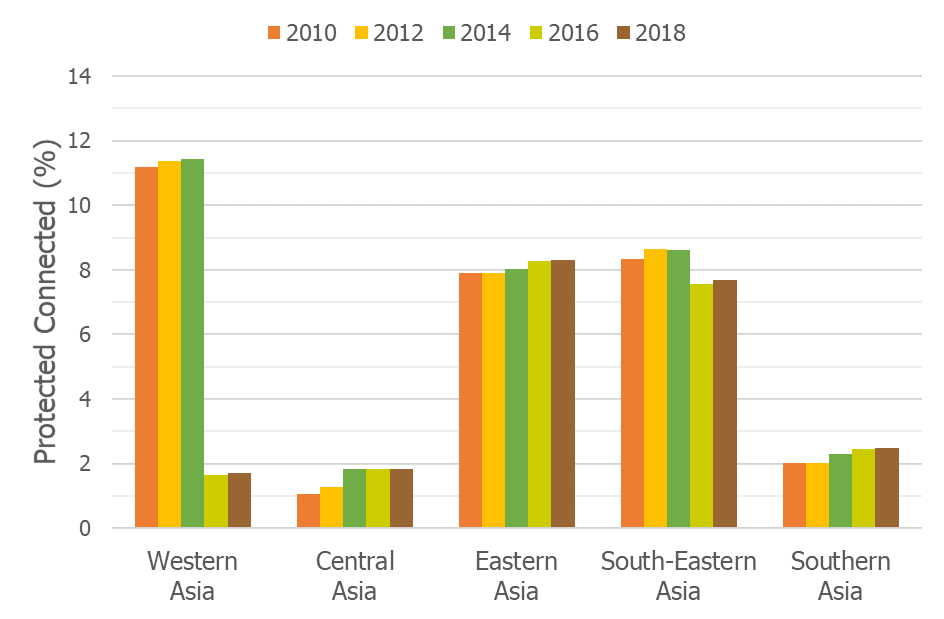
**

**Fig. B.3.** Trends in the percentage of protected and connected land (ProtConn) from 2010 to 2018 in the regions of Asia. ProtConn values correspond to ProtConn_Bound_, which focuses on the part of PA connectivity that is within the power of a country to influence, i.e. excluding PA isolation that is naturally imposed by the sea or that is due to foreign lands (Saura et al., 2018). Regions are defined according to the M49 standard of the United Nations Statistics Division (called sub-regions in M49). The ProtConn decrease in Western Asia was mainly due to PA degazettement in Oman and particularly in Saudi Arabia (Fig. 3). The expiration of a hunting ban in two very large (0.5 million km^2^) PAs in Saudi Arabia led to their removal from the WDPA in 2016 (Lewis et al., 2019).

**
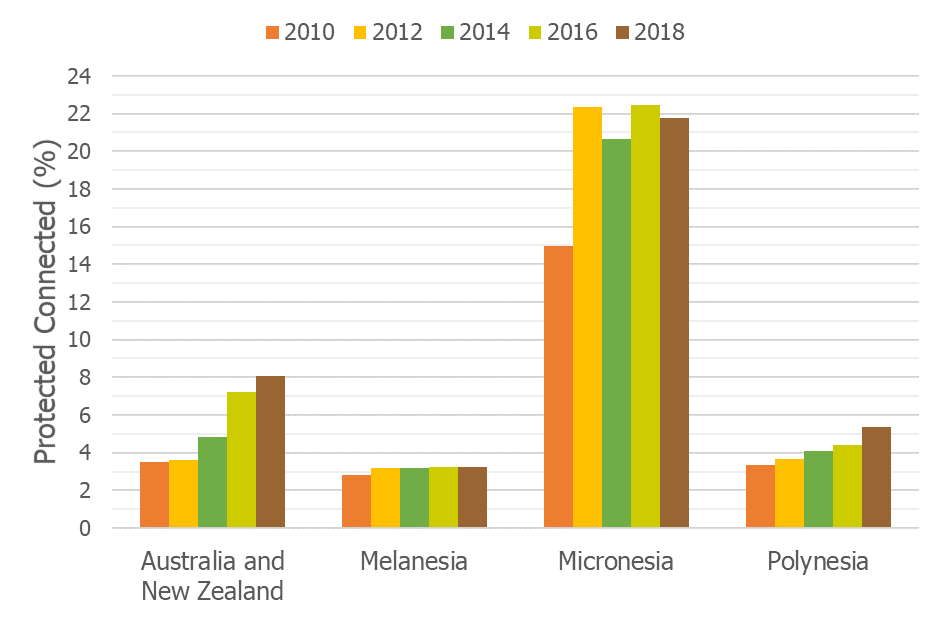
**

**Fig. B.4.** Trends in the percentage of protected and connected land (ProtConn) from 2010 to 2018 in the regions of Oceania. ProtConn values correspond to ProtConn_Bound_, which focuses on the part of PA connectivity that is within the power of a country to influence, i.e. excluding PA isolation that is naturally imposed by the sea or that is due to foreign lands (Saura et al., 2018). Regions are defined according to the M49 standard of the United Nations Statistics Division (called sub-regions in M49).

**
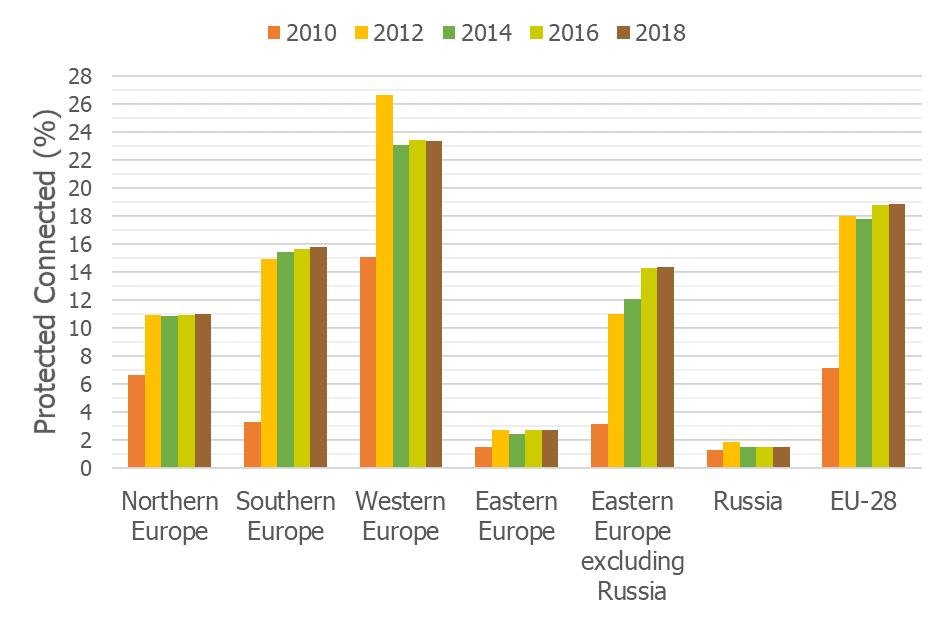
**

**Fig. B.5.** Trends in the percentage of protected and connected land (ProtConn) from 2010 to 2018 in the regions of Europe. Regions are defined according to the M49 standard of the United Nations Statistics Division (called sub-regions in M49). ProtConn values correspond to ProtConn_Bound_, which focuses on the part of PA connectivity that is within the power of a country to influence, i.e. excluding PA isolation that is naturally imposed by the sea or that is due to foreign lands (Saura et al., 2018). The regional values are influenced by how countries are grouped into regions according to this M49 standard. For instance, the Russian Federation is included within the region of Eastern Europe. This has a considerable effect on the values for Eastern Europe, given that the Russian Federation alone is larger than all other Eastern European countries. For this reason, the figure provides the ProtConn values separately for the Russian Federation and for the rest of the Eastern European countries, in addition to the value for all the Eastern European countries together. The figure also gives the values for the EU, which were obtained considering the 28 countries which are currently part of the EU (EU-28), and excluding PAs and territories whose reported ISO3 in the WDPA was different from those 28 countries, even if under the sovereignty of a EU member state, as in the case for example of Réunion Island. For the EU, both Natura 2000 sites and nationally designated sites have been considered in this assessment.


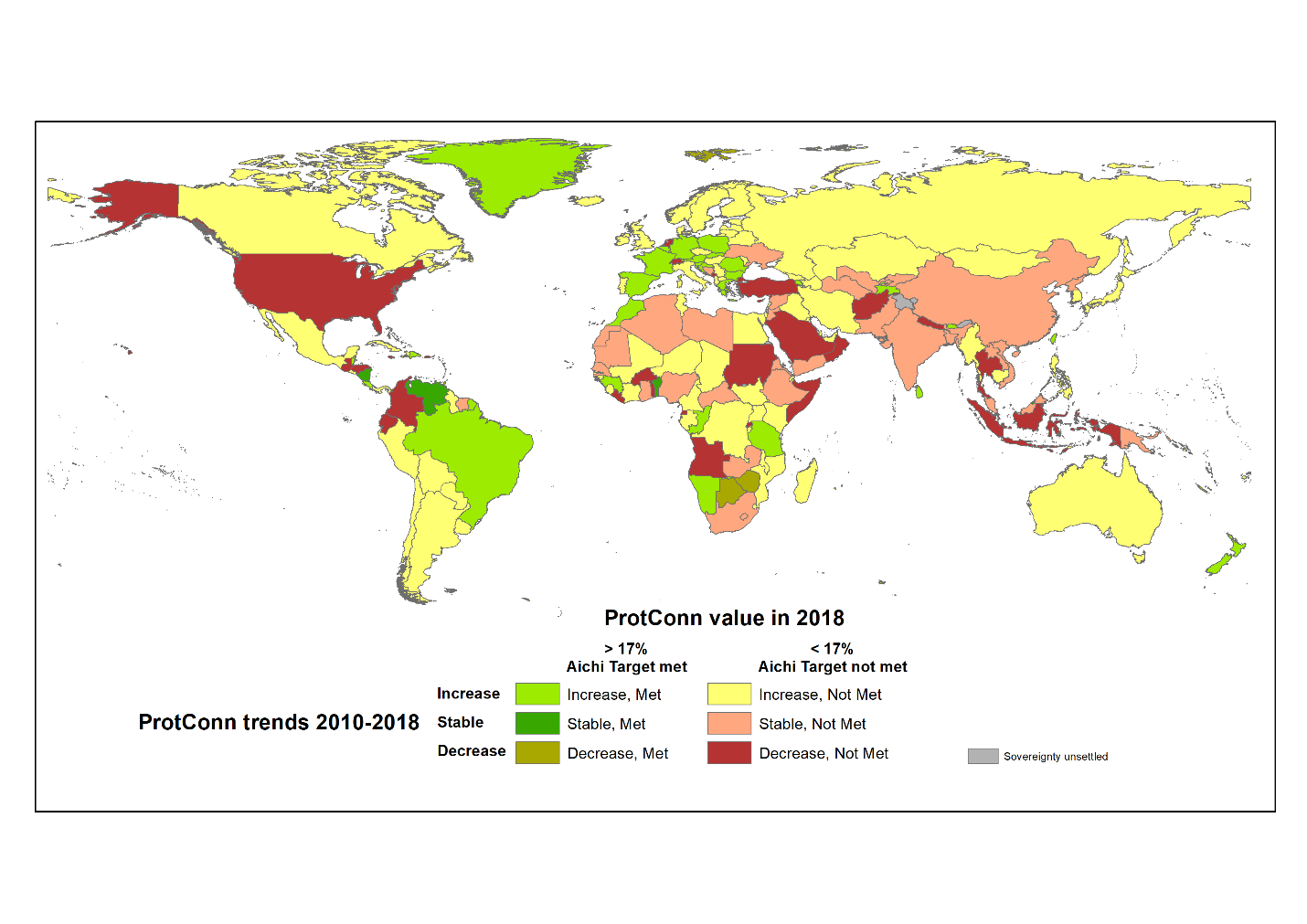


**Fig. B.6**. ProtConn trends (stable, increasing or decreasing) for all countries from 2010 to 2018 using a threshold of 0.1% for defining the stable class, and current status of the countries (as of 2018) regarding the connectivity element of Aichi Target 11, which is assumed to be met if ProtConn≥17%. Countries with a stable trend have less than 0.1% of absolute difference in their ProtConn values in 2010 and 2018. India, for example, had a ProtConn of 1.15% in 2010 and a very similar ProtConn of 1.19% in 2018. ProtConn values correspond to ProtConn_Bound_, which focuses on the part of PA connectivity that is within the power of a country to influence, i.e. excluding PA isolation that is naturally imposed by the sea or that is due to foreign lands (Saura et al., 2018).


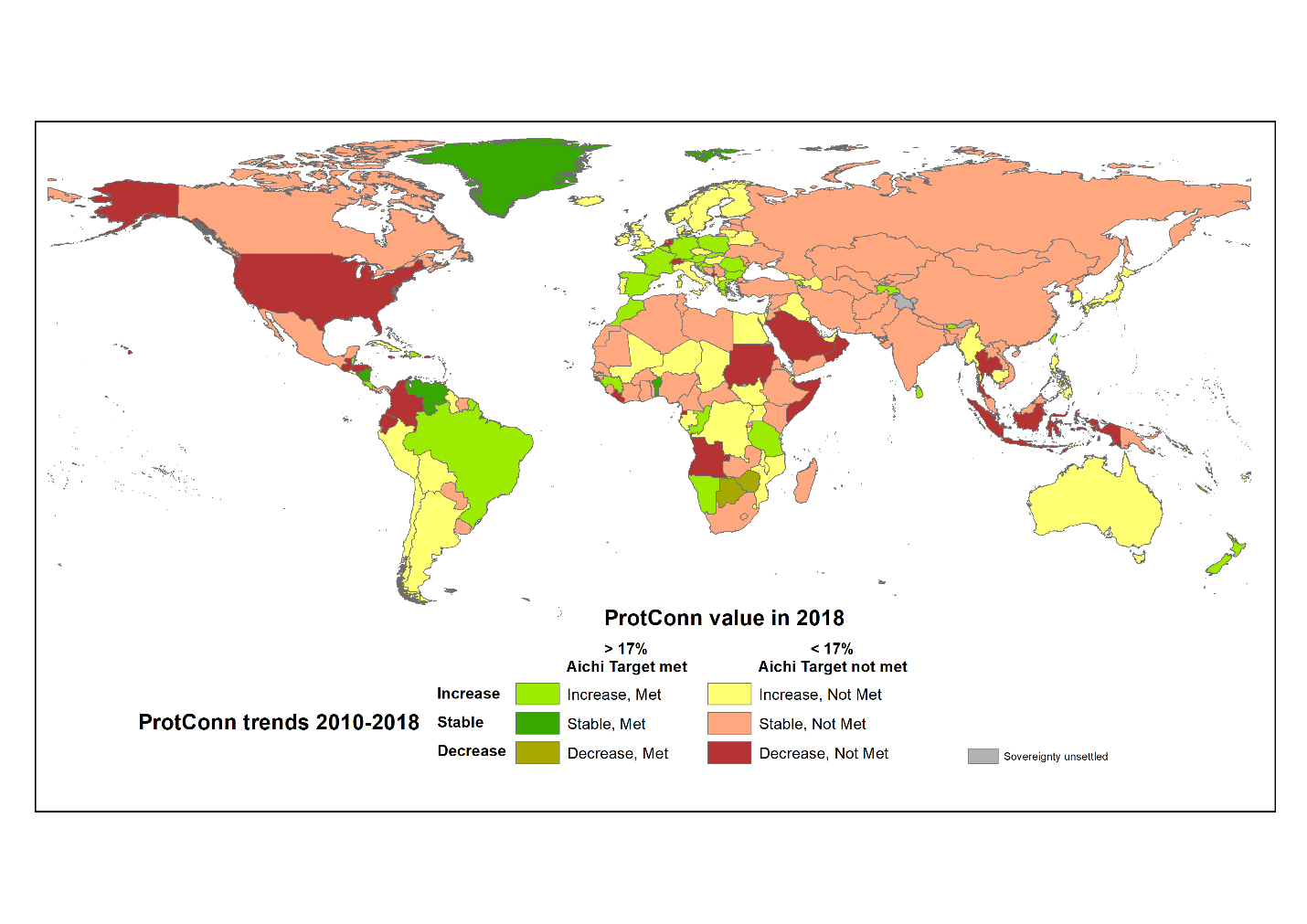


**Fig. B.7**. ProtConn trends (stable, increasing or decreasing) for all countries from 2010 to 2018 using a threshold of 0.5% for defining the stable class, and current status of the countries (as of 2018) regarding the connectivity element of Aichi Target 11, which is assumed to be met if ProtConn≥17%. Countries with a stable trend have less than 0.5% of absolute difference in their ProtConn values in 2010 and 2018. India, for example, had a ProtConn of 1.15% in 2010 and a very similar ProtConn of 1.19% in 2018. ProtConn values correspond to ProtConn_Bound_, which focuses on the part of PA connectivity that is within the power of a country to influence, i.e. excluding PA isolation that is naturally imposed by the sea or that is due to foreign lands (Saura et al., 2018).


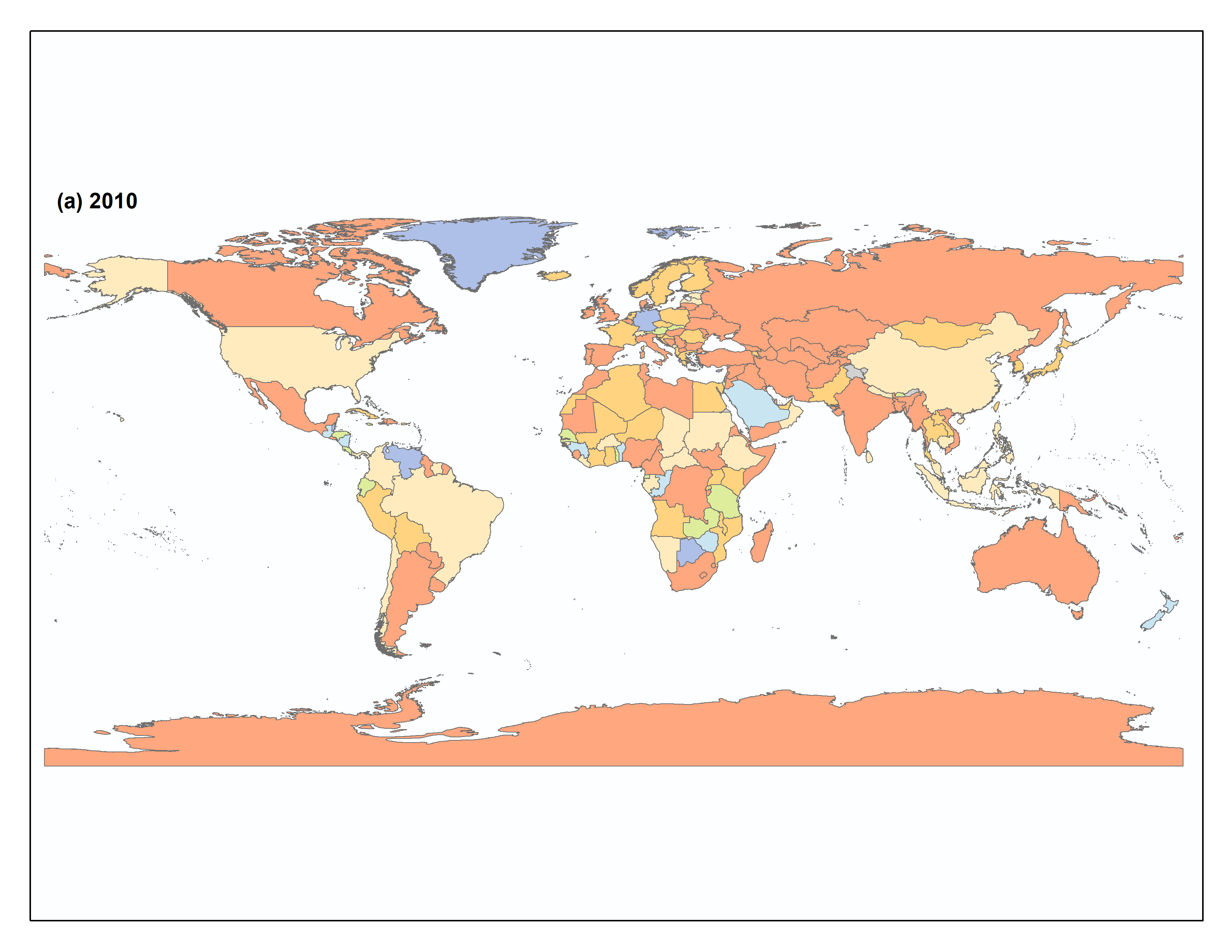


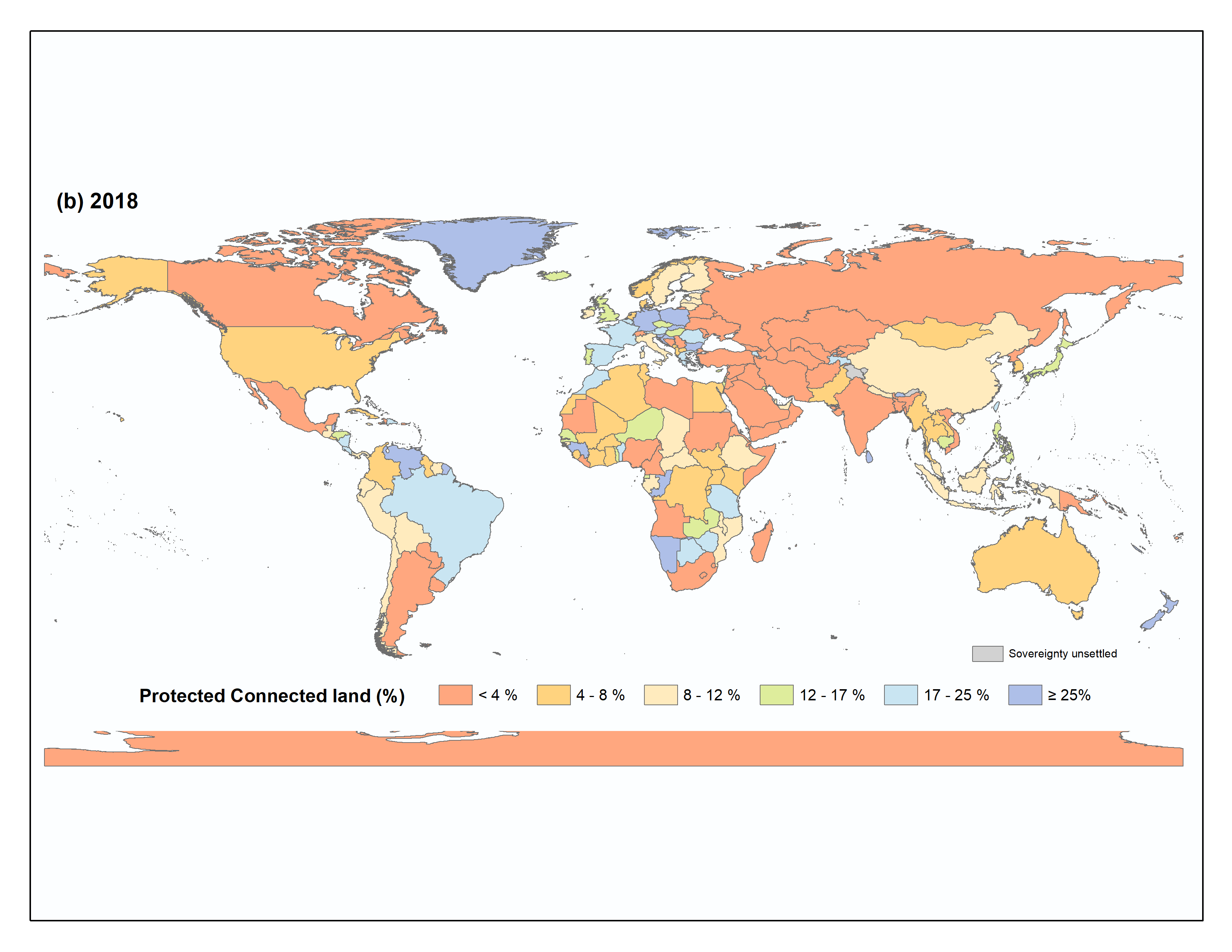


**Fig. B.8**. Percentage of Protected and Connected land (ProtConn) for all countries as of 2010 (a) and 2018 (b). The two blue classes include the countries that meet in 2010 (a) or in 2018 (b) the Aichi Target 11 element on connectivity, which is assumed to be met if ProtConn≥17%. ProtConn values correspond to ProtConn_Bound_, which focuses on the part of PA connectivity that is within the power of a country to influence, i.e. excluding PA isolation that is naturally imposed by the sea or that is due to foreign lands (Saura et al., 2018).


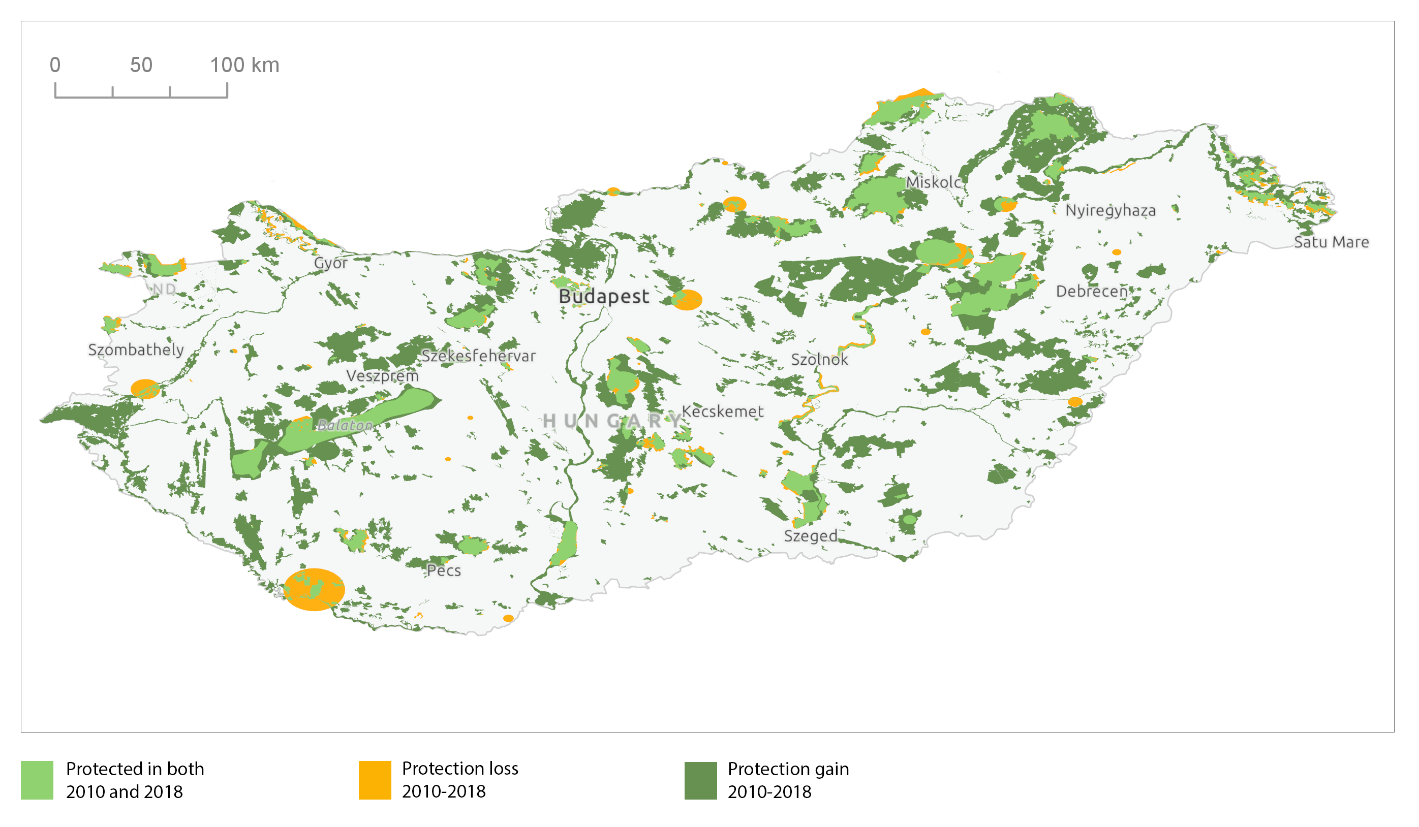


**Fig. B.9**. Distribution of protected areas (PAs) in 2010 and 2018 in Hungary, a country that experienced a remarkable increase in ProtConn in that period, from 2.2% in 2010 to 15.4% in 2018. The PA layers correspond to the dissolved PA layers used in this study, which do not show the boundaries between adjacent or overlapping PAs nor the PA polygons smaller than 1 km^2^.

1. <http://www.fao.org/geonetwork/srv/en/metadata.show?id=12691> [↑](#footnote-ref-1)
2. <https://www.protectedplanet.net/c/monthly-updates/2018/may-2018-update-of-the-wdpa> [↑](#footnote-ref-2)
3. Available at <https://www.protectedplanet.net/> [↑](#footnote-ref-3)
